# State-of-the-art combination treatment strategies for advanced stage non–small cell lung cancer

**DOI:** 10.3389/fonc.2022.958505

**Published:** 2022-08-01

**Authors:** Yongfang Yao, Rameesha Fareed, Aliya Zafar, Kalsoom Saleem, Tao Huang, Yongtao Duan, Masood Ur Rehman

**Affiliations:** ^1^ Henan Provincial Key Laboratory of Children’s Genetics and Metabolic Diseases, Children’s Hospital Affiliated to Zhengzhou University, Zhengzhou University, Zhengzhou, China; ^2^ Key Laboratory of Advanced Drug Preparation Technologies, Ministry of Education, School of Pharmaceutical Sciences, Zhengzhou University, Zhengzhou, China; ^3^ State Key Laboratory of Esophageal Cancer Prevention and Treatment, School of Pharmaceutical Sciences, Zhengzhou University, Zhengzhou, China; ^4^ Riphah Institute of Pharmaceutical Sciences, Riphah International University, Islamabad, Pakistan; ^5^ Medical School, Huanghe Science and Technology University, Zhengzhou, China

**Keywords:** lung cancer, small cell lung cancer, advanced stage, chemotherapy, immunotherapy, targeted therapy

## Abstract

Non–small cell lung cancer (NSCLC) is the most abundant type of epithelial lung cancer being diagnosed after 40% of invasions of excrescence in pulmonary tissues. According to WHO, 30% of NSCLC patients can be cured if diagnosed and treated early. Mutations play an important role in advanced stage NSCLC treatment, which includes critical proteins necessary for cellular growth and replication. Restricting such mutations may improve survival in lung cancer patients. Newer technologies include endoscopic bronchial ultrasonography and esophageal ultrasonography. Currently, policymaking or decision-making for treatment regimens merely depends on the genomic alterations and mutations. DNA sequencing, methylation, protein, and fragmented DNA analysis do NSCLC screening. Achievement of these goals requires consideration of available therapeutics in current anticancer approaches for improving quality of life and treatment outcomes for NSCLC patient. The specific goals of this review are to discuss first-line and second-line therapies for advanced-stage NSCLC and molecularly targeted therapy including thoughtful discussion on precise role of treatment strategies in specific tumors. Also, concerned diagnostics, new clinical trial designs, and pursuing appropriate combinations of radiotherapy and/or chemotherapy with biological therapy for exceptional cases considering resistance mechanisms and palliative care will be discussed.

## Introduction

Lung cancer has become one of the most widespread and deadliest cancers worldwide with non–small cell lung cancer (NSCLC) as predominating of all lung cancers, approximately 85% lower survival rate (1–6). An insignificant number of patients are detected at initial stage, including 26% and 8% at stages I and II, respectively, whereas later stages, like stage III and stage IV, are diagnosed more as 28% and 38%, respectively (7). It has been expected that ^2^/_3_ of NSCLC is usually on superior tiers of cancer III and IV while they may be diagnosed (8).

**Figure 1:**
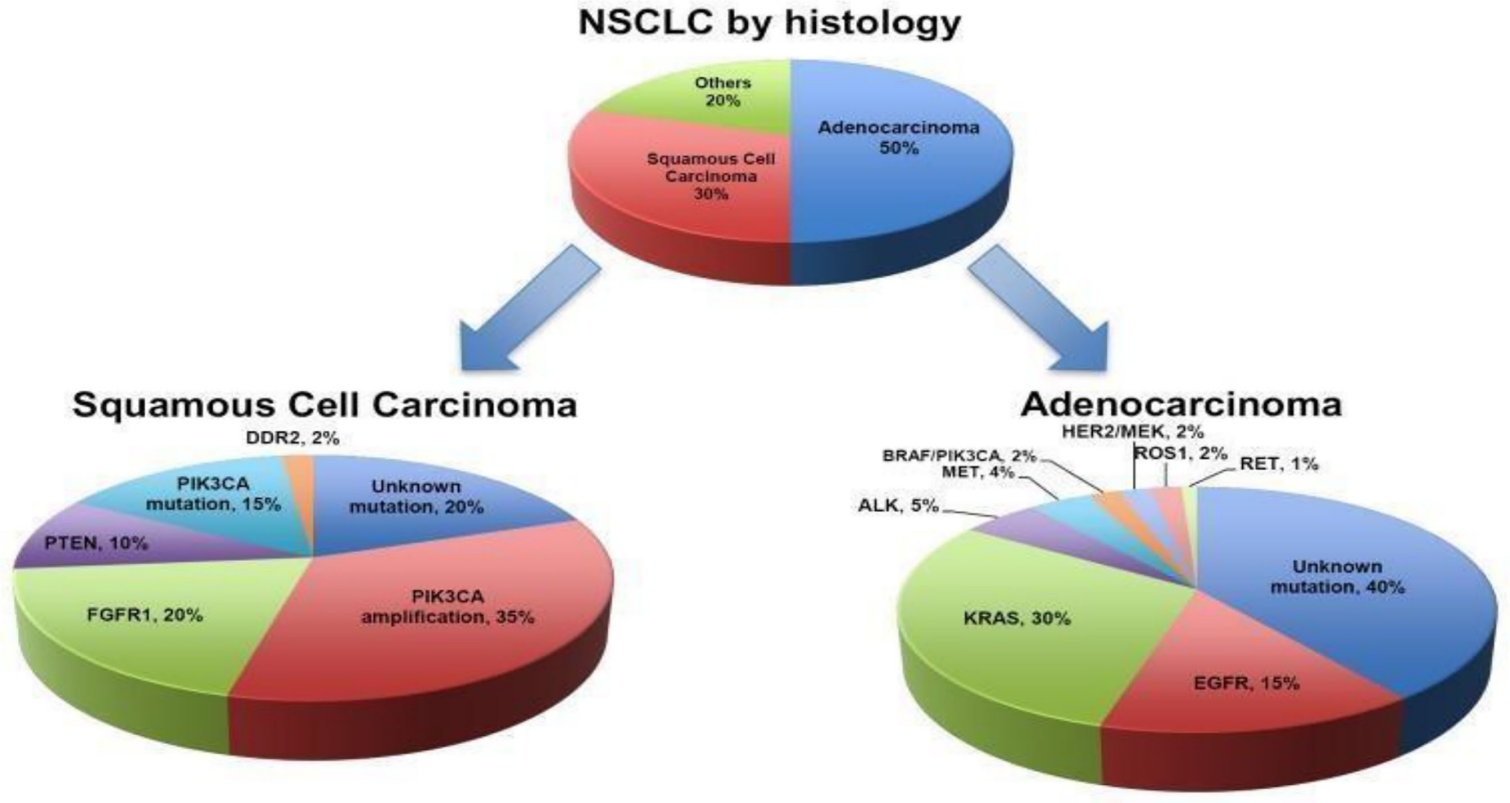
Classification of NSCLC (4).

Lung cancer classification, 2021, by World Health Organization (WHO) is based on histopathological and molecular subtypes into the following categories: precursor glandular and squamous precursor lesions, squamous cell, adeno-, adeno-squamous, large-cell, and sarcomatoid carcinomas; lung neuroendocrine neoplasm, tumors, and carcinomas; and salivary gland–type tumors (7) (**Figure 1**). For advanced NSCLC (9), owing to metastasis of the disease, NSCLC is rather aggressive and metastasizes early, involving the liver and brain, and is characterized by rapid tumor growth (10).

The survival rate for NSCLC is comparatively lower than other cancers and was approximately 16.8% and 25.1% for men and women, respectively, from the period of 2012 to 2015. Comparatively, the slow survival rate is because most NSCLC cases, about two-thirds, are detected at later stage or at unresectable IIIB and IV stages (3). For NSCLC, a 5-year survival rate is 25% influenced by multifarious factors, i.e., subtype and disease progress.

Advanced-stage NSCLC is characterized by metastases and is non-treatable with surgical resection if multiple metastatic sites are present. Patients with a single metastatic site are candidates for surgical removal of primary tumor. However, chemotherapy is frontline treatment for most advanced cases of NSCLC. Other two most common treatment options are either radiotherapy or palliative chemotherapy (3). Consistent exposure of lung epithelium to carcinogens leads to dysplasia and, if this persists, mutations arise and altered proteins will be synthesized resulting in disruption of cell cycle and sets stage for carcinogenesis. Genetic mutations most commonly responsible for pathogenesis of NSCLC are epidermal growth factor (EGFR), Kirsten rat sarcoma virus (KRAS), and p16 (7). Furthermore, greater risk of pulmonary embolism (PE) and thromboprophylaxis is associated with surgery. Current standard of care is concurrent chemoradiotherapy followed by immunotherapy (11).

## Current treatment strategies of advanced-stage NSCLC

The goal of treating advanced stages of NSCLC is to improve and prolong patient’s life and alleviate symptoms. Cancer stage consideration is important for determining treatment choices of NSCLC. According to the Union of International Cancer Control (UICC)/American Joint Committee on Cancer (AJCC), TNM’s classification has been considered as the golden standard for staging and subsequent prognosis of solid tumors. The latest guidelines of the eight TNM staging edition recognize multi-model therapy crucial for unresectable stage III NSCLC, considerable superior effects of chemoradiotherapy followed by durvalumab [anti–programmed death ligand 1 (PD-L1) agent], approved by United States Food and Drug Administration (US FDA) over standard chemoradiotherapy (12). Treatment options for stages I and IIA and IIB are surgery and, later, adjuvant chemotherapy. Chemotherapeutics used in NSCLC include primarily platinum analogs (cisplatin and carboplatin) along with mitomycin C, ifosfamide, and vinca alkaloids (vindesine, vinorelbine, and vinblastine), as well as etoposide, gemcitabine, pemetrexed, and taxanes such as paclitaxel and docetaxel (13).

## Surgery in advanced-stage NSCLC

Stage III is the most heterogeneous stage due to tumor invasion and involvement of lymph nodes, and therefore, patients are considered for a multidisciplinary treatment approach (14). At stage 0, surgery is the most fruitful treatment option because, at this stage, tumor is neither invasive nor metastatic in nature; segmentectomy is beneficial in this case, while, in case of centrally located lesions, either lobectomy or endobronchial therapy is performed, including electrodynamics therapy, cryotherapy, ND-YAG laser therapy, and electrocautery.

At stages IA and IB, an evidence-based study showed a 4-year survival outcome with lobectomy with complete ipsilateral mediastinal lymph node dissection (CMLND) in comparison to lymph node sampling. This currently holds limitations and rejection as the treatment of choice because of reduced efficacy at all stages. Therapeutic surgery is regarded as the treatment of choice for stage IIIA with N1 lymph nodes. However, a large number of patients are diagnosed with N2 disease (14). Therefore, surgery is followed by adjuvant chemotherapy in current consensus. For stage IIIA1 and IIIA2 patients, a mediastinal lymphadenectomy is often followed by platinum-based adjuvant chemotherapy (5).

## First-line systemic treatment in metastasized NSCLC

**Figure 2:**
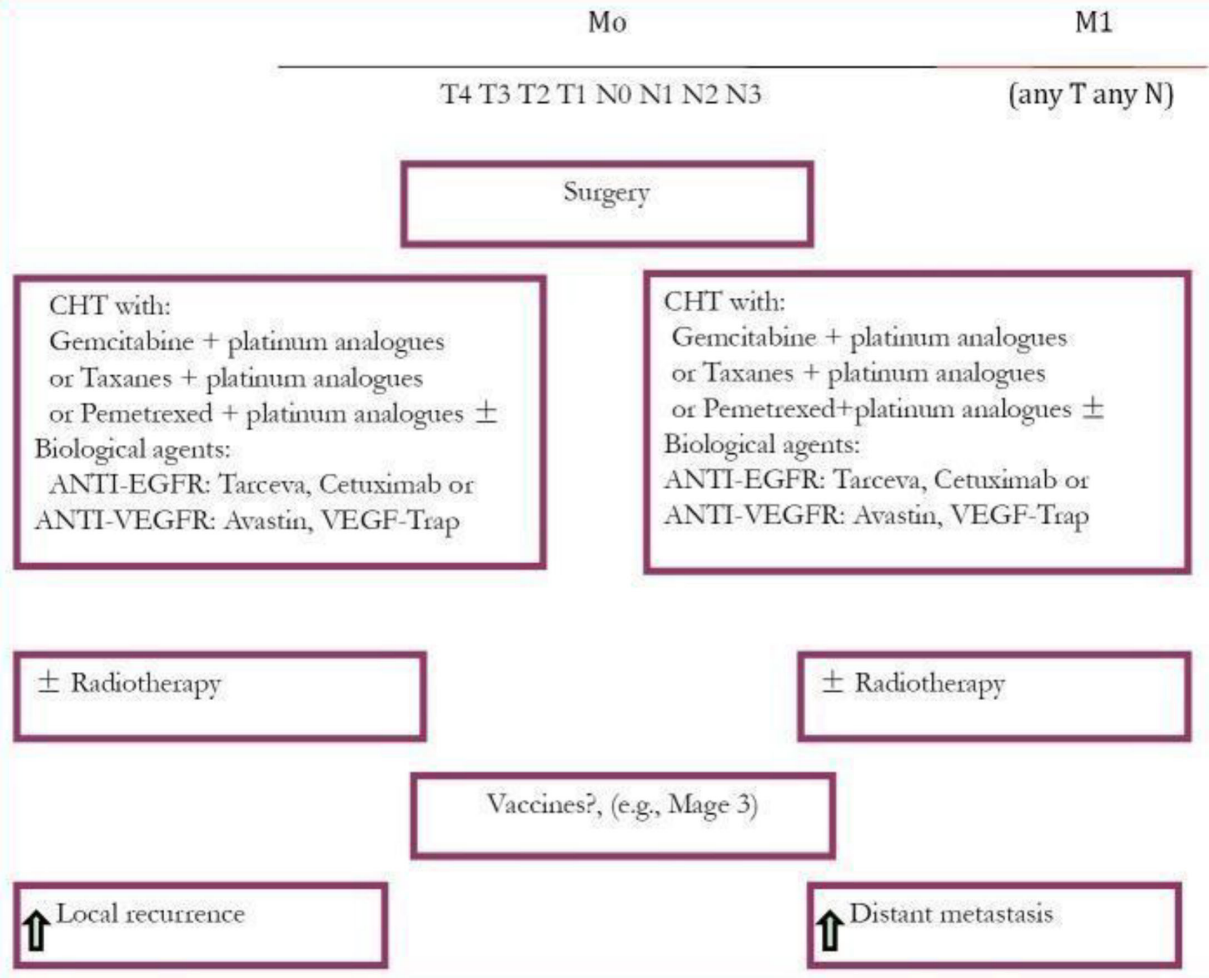
Treatment plan for NSCLC (13).

Postoperative radiation therapy (PORT) is not evidenced as effective in stage I patients and did not improve survival rate. The restricting mutations may improve survival in lung cancer patients. These mutations are EGFR and anaplastic lymphoma kinase (ALK). EGFR is inhibited by tyrosine kinase inhibitors (TKIs) (**Figure 2**). Novel research in targeted drug therapy showed significant survival benefits to NSCLC patients up until stage IIIA, with EGFRs-sensitizing mutation like ceritinib (80 mg) improved disease-free survival.

**Figure 3:**
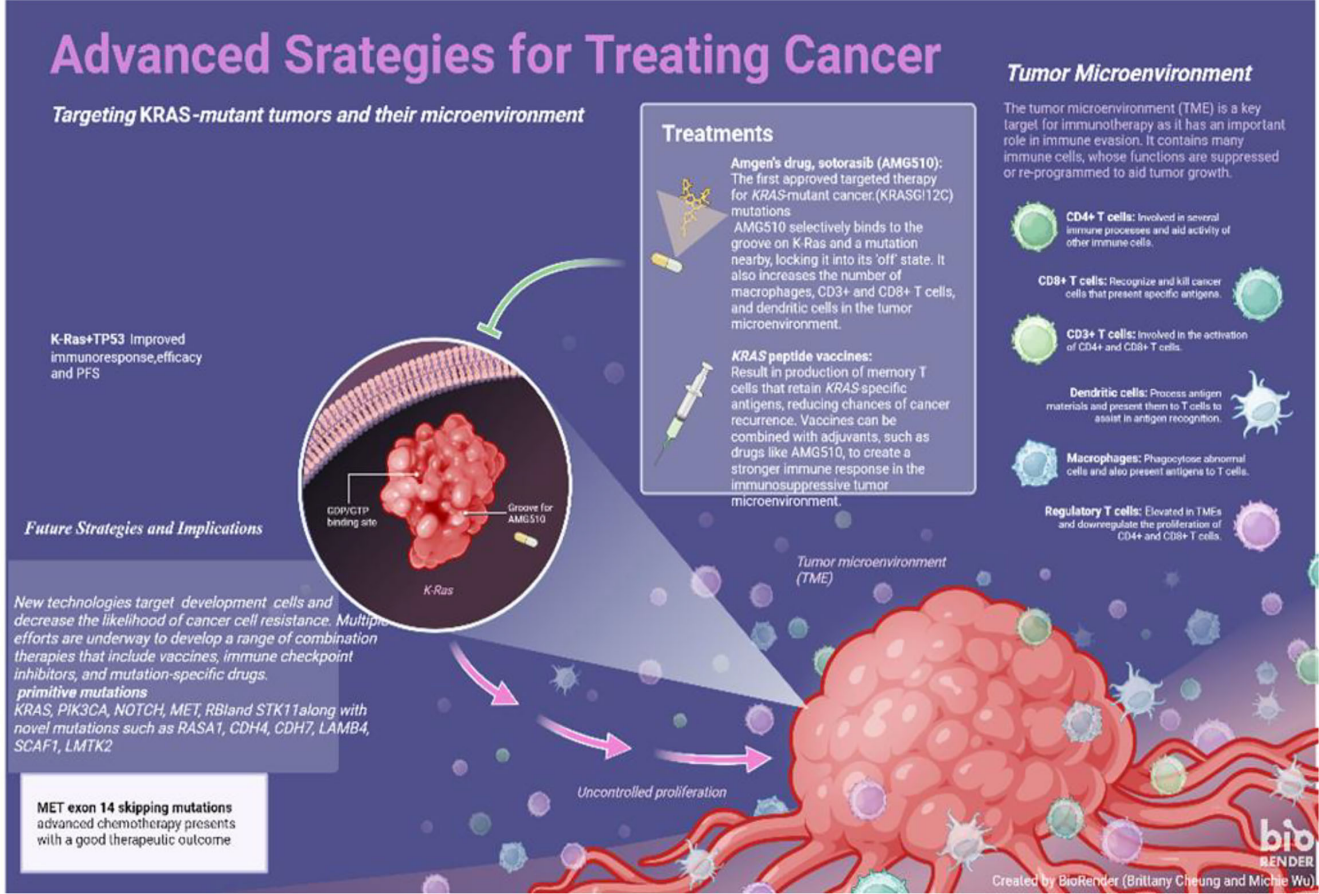
Advanced strategies for treating cancer.

Currently, standard for frontline treatment of advanced-stage NSCLC, which is negative for a mutant EGFR or ALK, is platinum-based doublet chemotherapy (PT-DC). Improved clinical outcomes have been observed by incorporation of bevacizumab (Bev) to first-line PT-DC, as compared to chemotherapy alone to treat non-squamous NSCLC (**Figure 3**).

In case of stage IIIA and IIIA4 patients, with tumors and N2/N3 lymph nodes, conditionally being healthy patient with no weight loss, best outcomes can be achieved with concurrent chemoradiotherapy followed by surgery including platinum-based chemoradiotherapy; however, this may cause severe esophagitis. To reduce local relapse of tumor, PORT can be used without prolonging survival. Nevertheless, meta-analysis of five randomized controlled trails (RCT) of cisplatin-based therapy has resulted in survival benefit (HR for death −0.89, 99% CI; 0.82–0.96) (13, 15).

Stage IV remains incurable and therapy aims at improving quality of life and survival of patient (12, 15). During this stage, only a small percentage of patients, 10%–30%, respond to chemotherapy and few, 1%–3%, survive 5 years after being diagnosed. Functional patients are offered double drug-based chemotherapy for small survival benefit (15). Numerous randomized trials demonstrated an overall survival (OS) benefit, with more than a hundred patients, by deploying chemotherapy treatment along with best supportive care (BSC) (13).

In elderly population, combination chemotherapy of gemcitabine and vinorelbine failed to show improvement in response rate, TTP, and quality of life. However, this combination has been well tolerated and effective in numerous phase II trials (16). Cisplatin- and carboplatin-based therapies have shown to be tolerable in geriatric population according to retrospective analyses of phase III RCT and multiple phase II studies. Weekly paclitaxel and carboplatin showed improvement in all outcome parameters in comparison to either single-agent vinorelbine or gemcitabine in randomized phase III trials (17). Overall response rate (ORR), survival benefit, progression-unfastened survival (PFS), median survival, and 1-year OS improved with paclitaxel and carboplatin (18). Clinically, non-platinum monotherapy is the first-line treatment for unfit geriatric patients with advanced NSCLC. Those who are physically fit enough have a better option of a carboplatin-based combination. More improvement in survival was observed with combination of Bev and paclitaxel/carboplatin (PCB) as compared to chemotherapy alone. However, this benefit was missing in women over the age of 60 (19, 20). According to two randomized phase III studies, i.e., the ECOG 4599 and AvAil, incorporation of Bev, an anti-angiogenic agent, to carboplatin and paclitaxel regimen in the first study and gemcitabine/cisplatin in the second study improved effectiveness and PFS from 4.5 to 6.2 months, P < 0.0001. In comparison to control arm, the arm receiving Bev in ECOG 4599 study had improved OS statistically (HR 0.79; 95%, CI: 0.67–0.92; P = 0.003) (21). Hence, results of these two trials indicated that Bev can be recommended to be used in combination with chemotherapy in NSCLC treatment (13).

## Pemetrexed-platinum doublet chemotherapy with or without bevacizumab

Pemetrexed-platinum doublet (Pem-Pt) is a combination of platinum-based chemotherapy with Bev and is regarded as category 1 regimen for advanced-stage NSCLC. The addition of Bev to Pem-Pt doublet regimen exhibited longer median PFS and higher ORR in general population (P = 0.000). The addition of Bev as maintenance therapy after Pem-Pt plus Bev regimen demonstrated a longer median PFS in comparison to patients without Bev in maintenance therapy. The Pem-Pt plus Bev regimen was associated with an acceptable safety profile, which lacked incidences of hypertension, proteinuria, or excessive bleeding (6) (**Table 1**). Bev is approved along with chemotherapy in advanced non-squamous NSCLC owing to its antiangiogenic effects, anti–vascular endothelial growth factor (VEGF), and immunomodulatory effects. It enhances efficacy of atezolizumab to reverse VEGF immunosuppression (29).

**Table 1:** Chemotherapeutics agents in maintenance designed trials.

Trial	Number randomized	First-line agents	Maintenance	Survival in months (Hazard ratio; P-value)
(22)	181	MIC	Vinorelbin	12.3vs.12,3 (HR=1.08;P=0.48)
(23)	206	GC	Gemicitabine	OS13vs.11 (HR=n.r;P=0.195)
(24)	464	GC	Gemicitabine	PFS 3.7vs.2.1 (HR=0.51;P<0.001)
(25)	255	GCb	Gemicitabine	OS 8vs.9.3 (HR=0.97,P=0.84)
(26)	307	GCb	Docetaxel	OS12.3 vs.9.7 (HR=n.r;P=0.0853)
(27)	663	Cb/CG/Pac/D	Pemetrexed	OS13.4 vs.10.6 (HR=0.79; P=0.012)
(28)	539	PemC	Pemetrexed	PFS3.9vs.2.6 (HR=0.64; P=0.002)

In a more recent trial, BeTa (Bev/Tarceva) trial, a combination of Bev and erlotinib, as second-line treatment of advanced-stage NSCLC was investigated. Results suggested that the combination doubled PFS (3.4 months) in comparison to monotherapy of erlotinib alone (1.7 months, P = 0.001) with no improvement regarding OS (13). Other randomized trial of targeted therapy suggested that addition of Bev enhances chemotherapeutic response but no increment in OS (13).

## Nivolumab monotherapy as first-line treatment of advanced-stage NSCLC

Nivolumab is an antibody that targets programmed cell death protein 1 (PD-1) as an immune checkpoint inhibitor (ICI). It is shown to improve ORR of 17% in heavily pretreated patients of advanced NSCLC and 1- to 3-year OS rates of 42%, 24%, and 18%, respectively, evidenced through phase I, multi-cohort, and checkmate 0.12 trial. Previous study conducted on 423 patients reported >5% PD-L1 expression and minimal advantage of nivolumab treatment in RR (26% vs. 33%), PFS (4.2 vs. 5.9 months; HR, 1.15; 95% CI, 0.91–1.45; P = .25), or OS (14.4 vs. 13.2 months; HR, 1.02; 95% Q17 CI, 0.80–1.30) were observed (30).

## Nivolumab and ipilimumab in combination and monotherapy

Nivolumab is of particular importance and improved survivors to chemotherapy coupled with low toxicity profile and survival rate. It is used as second-line monotherapy for squamous and non-squamous cell metastatic NSCLC. Phase III trial (CheckMate 227) studied the combination therapy of nivolumab and ipilimumab (ICI antibodies) in previously untreated patients of advanced-stage NSCLC (31, 32). PD-1 and PD-L1 inhibitors are currently recommended second-line remedies apart from first-line remedies (33).

Disease progression-free survival was reported to be less than 1% PD-L1 with nivolumab plus chemotherapy versus chemotherapy alone in the first part of the study. Median PFS was observed for 5.6 months with nivolumab and chemotherapy and 4.7 months without nivolumab (95% CI: 0.58–0.94; HR: 0.74) (31). Results of phase II CheckMate 568 trials emphasized that increased TMB, which are not associated with PD-L1 status, showed better response [PD-L1 and tumor mutational burden (TMB); predictors of response to immunotherapy].

## Atezolizumab plus chemotherapy and bevacizumab as first-line treatment for advanced-stage NSCLC

Incorporation of atezolizumab to a regimen consisting of Bev and chemotherapy significantly improves PFS in advanced non-squamous NSCLC (34). This result is not influenced by expression of EGFR or ALK. The promising efficacy and reasonable safety profile increased when combined with a platinum doublet chemotherapy in NSCLC cases not treated with chemotherapy before (35).

## Monotherapy or combination of ICI as first-line treatment for advanced NSCLC

Incorporation of ICIs shows sustainable anti-tumor activity and increases long-term survival (36). Chemotherapy along with pembrolizumab and then with atezolizumab demonstrated much greater response in comparison to all treatments, for both non-squamous and squamous patients. In non-squamous histology, combining chemotherapy with pembrolizumab and atezolizumab/Bev chemotherapy, seconded with pembrolizumab monotherapy and atezolizumab chemotherapy, has shown to be the best treatment generally in overall cohort (37). Pembrolizumab is being used as a first-line regimen for advanced-stage NSCLC with a PD-L1 expression of ≥50%. On this context, necitumumab along with platinum-based chemotherapy can become an affordable substitute as a first-line treatment protocol. In squamous NSCLC with PD-L1 expression <50%, quadruple schedules of platinum doublet plus pembrolizumab and necitumumab are being used (8).

## Second-line agents for advanced-stage NSCLC

The standard second-line agents include pemetrexed, docetaxel, erlotinib, and gefitinib (TKIs). In patients who carry a mutant EGFR, the TKIs are preferred the second-line agent if not used in the first-line therapy. Crizotinib, a newly FDA-approved drug for ROS-1 mutation expressing cancers (36), is an EML4/ALK fusion protein inhibitor (36). MET/ALK inhibitor, crizotinib, is under clinical trials along with a pan-HER inhibitor (dacomitinib). Among ALK inhibitors are ceritinib and alectinib, and crizotinib was granted an FDA approval in 2011 and proved to be superior to second-line chemotherapy in patients who already received platinum doublet with a median PFS of 7.7 months with crizotinib as compared to docetaxel or pemetrexed chemotherapy with PFS of 3 months (HR 0.49; 95% CI, 0.37–0.64, P < 0.001) and, therefore, showed greater survival advantage in comparison to patients not receiving crizotinib. It is orally active and works as a small-molecule inhibitor of ALK, MET, and ROS tyrosine kinases (4). Currently, crizotinib is being evaluated as a first-line agent over platinum-pemetrexed chemotherapy in phase III PROFILE 1014 study to treat ALK-positive NSCLC (**Table 2**). With the NSCLC harboring ALK or ROS1 rearrangements, RET-rearranged lung cancers can respond to pemetrexed-based doublet chemotherapy with an ORR of 45% and PFS of 19 months (38, 39). Erlotinib, another EGFR inhibitor, has shown promising results in randomized phase III trials primarily as second-line and third-line therapy, maintenance therapy, and in patients carrying mutations in EGFR. Brigatinib is first line against crizotinib in advanced ALK+NSCLC and was shown to have better activity than crizotinib in ALTA-1L trial [52, 53]. Another first-line agent, lolatinib, was evaluated against crizotinib (phase III randomized CROWN trial) and resulting ORR was 76%, which was higher than 58% of the crizotinib group [51] [13, 35] (**Table 3**).

**Table 2:** FDA approved targeted agents for advanced NSCLC (1).

Actionable mutation	FDA approved therapy (citation)	Clinical trial (phase)	Comparator	ORR (%)	mPFS (months)	mOS (months)	Adverse effects
KRAS	Sotorasib	CodeBreaK 100 (I)	No	32%	6.3	12.5	Diarrhea, nausea, elevated LFT’s, fatigue
EGFR	Erlotinib	EURTAC (III)	Chemotherapy	64%	9.7	22.9	Fatigue, rash, diarrhea
	Gefitinib	NEJ002 (III)	Carboplatin/Paclitaxel	74%	10.8	27.2	Rash, diarrhea
	Afatinib	LUX-Lung 3 (III)	Cis/Pemetrexed	56%	11.1	28.2	Rash, diarrhea, paronychia
	Dacomitnib	ARCHER 1050 (III)	Gefitinib	75%	14.7	34.1	Diarrhea, paronychia, rash
	Osimertinib	FLAURA (III)	Erlotinib/Gefitinib	80%	18.9	38.6	Rash, diarrhea, pneumonitis
ALK	Crizotinib	PROFILE 1014 (III)	Platinum/Pemetrexed	74%	10.9	NR	Vision disorder, diarrhea, edema
	Certinib	ASCEND-4 (III)	Platinum/Pemetrexed	73%	16.6	51.3	Diarrhea, nausea, vomiting
	Alectinib	ALEXALEX (III)	Crizotinib	83%	25.7	Immature	Elevated LFT’s, CPK elevation, anemia
	Brigatinib	ALTA 1L (III)	Crizotinib	74%	24	47.6	Elevated CPK and LFT’s
	Ensartinibǂ	eXALT-3 (III)	Crizotinib	75%	25.8	Immature	Rash, pruritis, edema
	Lorlatinib	B7461006 (III)	Crizotinib	76%	NR	Immature	Hyperlipidemia, edema, increased weight
MET Exon 14 skipping mutation	Capmatinib	GEOMETRY-mono-1 (II)	No	41% (68%) *	5.4 (12.4)*	NA/NA	Peripheral edema, nausea
	Tepotinib	VISION (II)	No	46%	8.5	Immature	Peripheral edema
MET amplification	Capmatinib	GEOMETRY-mono-1 (II)	No	29% (40%)*	4.1 (4.2)*	NA/NA	Peripheral edema, nausea
BRAF mutations	Dabrafenib + Trametinib	BRF113928 (II)	No	64% (68%)*	10.8	17.3	Pyrexia, LFT elevation, HTN
					(10.2)*	(18.2)*	
RET	Selparcatinib	LIBRETTO-001 (II)	No	64% (85%) *	16.5 (NR)	NR/NR	Dry mouth, diarrhea, HTN
	Pralsetinib	ARROW (II)	No	61%	16.5 (13)*	NA/NA	LFT elevation, anemia
				(70%)*			
ROS1	Crizotinib	PROFILE 1001 (I)	No	72.40%	19.3	51.4	Vision disorder, nausea, edema
	Certinib	NCT01964157(II)	No	62% (67%)*	9.3 (19.3)*	24	Diarrhea, nausea, anorexia
	Lorlatinib	NCT01970865 (I-II)	No	41% (62%)*	8.5	NA	Dyslipidemia
					(21)*		
	Entrectinib	STARTRK-1, STARTRK-2, ALKA-372–001	No	77%	19	NR	Weight gain, neutropenia
		(I-II)					
NTRK	Larotrectinib	LOXO-TRK-14001 (I-II)	No	70%	NA	NA	LFT elevation, neutropenia, anemia
	Entrectinib	ALKA, STARTRK-1, STARTRK-2 (I-II)	No	70%	NA	NA	Dysgeusia, constipation, fatigue
HER2	T-DM1ǂ	NCT02675829 (II)	No	44%	5	NA	Infusion reactions, thrombocytopenia
	T-DXdǂ	DESTINY-Lung01 (II)	No	62%	14	NA	Neutropenia, anemia, ILD

*Indicates data for treatment naïve patient.

**Table 3:** Effects of first-line and second-line treatments in NSCLC (67).

Overall Population		EGFR mutation		KRAS mutation		BRAF mutation		HER2 mutation		PIK3CA mutation		ALK rearrangement		Full WT
(n = 17664)		(n = 1,787)		(n = 4,588)		(n = 230)		(n = 92)		(n = 157)		(n = 340)		(n = 2,769)
		All	Adapted^$^	All	Adapted^$^	All	Adapted^$^	All	Adapted^$^	All	Adapted^$^	All	Adapted^$^	All
First-line Treatment														
Number with data %	8,448 (48%)	1,128 (63%)	662 (37%)	2,085 (45%)	979 (21%)	146 (64%)	64 (28%)	62 (67%)	28 (30%)	73 (47%)	29 (19%)	236 (69%)	120 (35%)	1,214 (44%)
Pemetrexed-based regimen	2,747 (33%)	188 (17%)	57 (9%)	792 (38%)	525 (54%)	51 (35%)	34 (53%)	31 (50%)	18 (64%)	17 (23%)	11 (38%)	111 (47%)	55 (46%)	401 (33%)
Vinorelbine-based regimen	504 (6%)	39 (3%)	9 (1%)	128 (6%)	68 (7%)	5 (4%)	2 (3%)	0	0	7 (10%)	3 (10%)	13 (6%)	9 (8%)	80 (7%)
Taxane-based regimen	1,064 (13%)	60 (5%)	18 (3%)	261 (13%)	166 (17%)	20 (14%)	12 (19%)	8 (13%)	4 (14%)	11 (15%)	7 (24%)	17 (7%)	11 (9%)	188 (16%)
EGFR-TKI	684 (8%)	543 (48%)	520 (79%)	26 (1%)	9 (1%)*	3 (2%)*	2 (3%)*	0	0	1 (1%)*	1 (3%)*	4 (2%)*	2 (12%)*	17 (1%)
Crizotinib	18 (<1%)	0	0	0	0	0	0	0	0	0	0	18 (8%)	18 (15%)	0
Trial^£^	253 (3%)	36 (3%)	31 (5%)	63 (3%)	48 (5%)	8 (6%)	5 (8%)	3 (5%)	1 (4%)	0	0	16 (7%)	12 (10%)	36 (3%)
Other^§^	709 (8%)	27 (2%)	9 (1%)	171 (8%)	77 (8%)	11 (8%)	3 (5%)	5 (8%)	3 (11%)	10 (14%)	5 (17%)	6 (3%)	3 (4%)	131 (11%)
BSC only	2,469 (29%)	235 (21%)	18 (3%)	644 (31%)	86 (9%)	48 (33%)	6 (9%)	15 (24%)	2 (7%)	27 (37%)	2 (7%)	51 (22%)	10 (8%)	361 (30%)
Second-line Treatment														
Number with data %	5,518 (31%)	698 (39%)	381 (21%)	1,358 (30%)	566 (12%)	106 (46%)	37 (16%)	43 (47%)	22 (24%)	48 (34%)	12 (8%)	157 (46%)	102 (30%)	797 (29%)
Taxane	782 (14%)	46 (7%)	34 (9%)	236 (17%)	203 (36%)	16 (15%)	8 (22%)	6 (14%)	4 (18%)	5 (10%)	2 (17%)	5 (3%)	4 (4%)	119 (15%)
Pemetrexed	612 (11%)	125 (18%)	97 (26%)	136 (10%)	105 (19%)	8 (8%)	6 (16%)	5 (12%)	4 (18%)	4 (8%)	2 (17%)	13 (8%)	10 (10%)	81 (10%)
Erlotinib	776 (14%)	231 (33%)	218 (57%)	125 (9%)	94 (17%)	9 (9%)	4 (11%)	5 (12%)	4 (18%)	2 (4%)	2 (17%)	10 (6%)	6 (6%)	96 (12%)
Crizotinib	73 (1%)	0	0	0	0	0	0	0	0	0	0	73 (46%)	73 (72%)	0
Trial^£^	116 (2%)	8 (1%)	7 (2%)	33 (2%)	27 (5%)	5 (5%)	5 (14%)	3 (7%)	2 (9%)	2 (4%)	1 (8%)	4 (3%)	4 (4%)	25 (3%)
Other^§^	442 (8%)	10 (1%)	6 (2%)	90 (7%)	60 (11%)	8 (8%)	7 (19%)	8 (18%)	8 (36%)	2 (4%)	2 (17%)	5 (3%)	3 (3%)	79 (10%)
BSC only	2,711 (49%)	272 (39%)	15 (4%)	738 (54%)	77 (14%)	60 (57%)	7 (19%)	16 (37%)	0	33 (69%)	3 (25%)	47 (30%)	2 (2%)	397 (50%)

*: Patients with tumors exhibited two molecular alterations including EGFR mutation. $: Selection of treatment based on the molecular analyses. £: Based on targeted therapy.

§: Including, but not limited to, another type of chemotherapy.

## Maintenance therapy in NSCLC

Maintenance therapy is treatment given to a patient after specific chemotherapy cycles when there is no disease progression and this is continued until either undesirable or toxic effects manifest or cancer progresses (41). Consolidation therapy is given following treatment with induction chemotherapy for specified number of cycles.

There are two ways to proceed with treatment: using an agent from induction regimen (continuation maintenance therapy) or incorporation of a different cytotoxic drug with different mechanism which was not included in first-line therapy known as the switch maintenance therapy (41). The drugs included as maintenance therapy in NSCLC are gemcitabine, docetaxel, and pemetrexed, and targeted agents include Bev, cetuximab, and erlotinib (41).

Maintenance therapy for NSCLC can be carried out in multiple ways, for example, continuation of induction therapy until disease progression, continuation of just non-platinum agents or molecularly targeted agent (continuation maintenance), and changing to another cytotoxic or molecularly targeted agent (switch maintenance). Randomized controlled trials show use of chemotherapy, immunotherapy, and molecularly targeted agents for maintenance therapy. The current standard treatment for advanced NSCLC comprises four–six sessions of a platinum-based doublet chemotherapy, which prolongs survival and alleviates symptoms. Another approach is administration of four sessions of cisplatin-based chemotherapy in combination with third-generation anti-EGFR or anti-VEGFR drug. It is not recommended to use platinum-based chemotherapy for advanced NSCLC for more than six cycles, according to ASCO guidelines (42).

## Maintenance chemotherapy after first-line therapy

## Trials on combination chemotherapy to treat advanced-stage NSCLC

The results of phase III trials of multidrug combinations including newer chemotherapeutics to treat advanced-stage NSCLC are discussed in **Table 1** that presents response of using third-generation cytotoxic drugs as monotherapy along with platinum analogs. Novel treatment with atezolizumab significantly increased disease-free survival (43). Adjuvant targeted therapy has the same results as it has at stage IA or IB.

At stage IIIA, surgical resection of the tumor and lymph node that it has spread to, with postoperative chemotherapy, is beneficial. While preoperative chemoradiation therapy may reduce tumor burden, chemoradiation therapy improves only disease-free survival DSF but not OS (13).

## Targeted therapy rationale

Targeted therapies consist of either small-molecule inhibitors or mAb or monoclonal antibodies (4). Targeted therapy for NSCLC rationale is based on targeting “driver mutations” which encode important proteins crucial for replication and cell growth. It is hypothesized that restricting mutations may improve survival at stage IVA (recurrent NSCLC).

Combination therapy, i.e., platinum therapy (cisplatin or carboplatin) in combination with paclitaxel, docetaxel gemcitabine, or premetrexed, is the treatment of choice (44). Addition of baclizumab (monoclonal antibody that targets endothelial vascular growth factor) to first-line treatment provides survival benefits.

**Figure 4:**
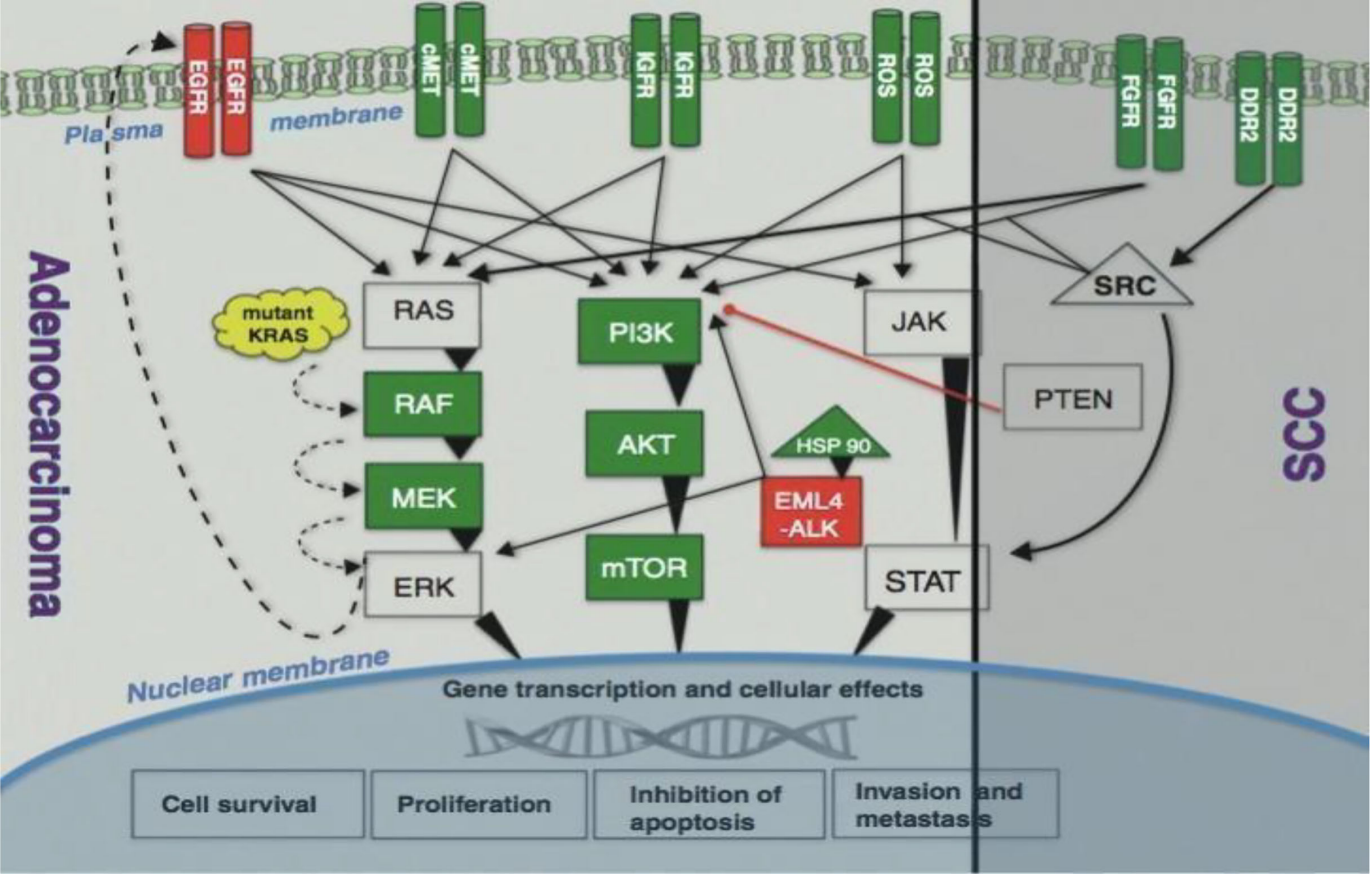
Signaling pathways (4).

For patients with EGFR-sensitizing mutations, EGFR TKIs are utilized that enhanced PSF profile of patient. Osimertinib is a drug of choice due EGFR and tyrosine kinase inhibition. Also, ALK inhibitors with ALK translocation, including crizotinib or alectinib, have greater PSF rate (45). The mutated protein kinases or receptors set off a cascade of signaling pathways such as PI3K-AKT-mTOR, MAPK or RAS-RAF-MEK-ERK, and JAK-STAT pathways, all of these play a major role in the uncontrolled growth and proliferation of tumor cells (**Figure 4**).

## Anti-EGFR monoclonal antibodies as targeted therapy

**Figure 5:**
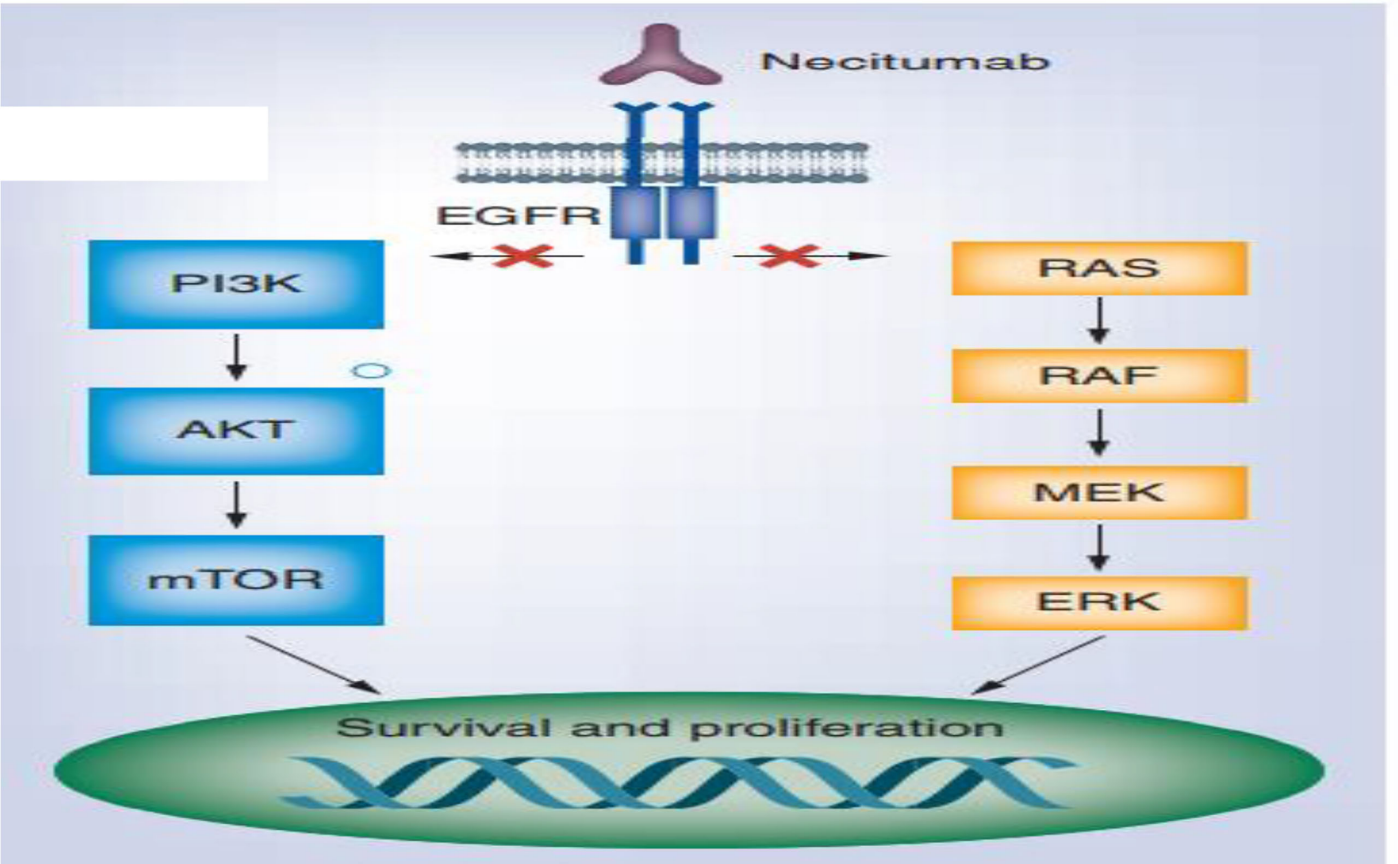
Necitumumab for the treatment of advanced (47).

Monoclonal antibodies targeting mutant EGFR and hinder receptor’s signaling through which bind to extracellular domain of receptor and form antibody-receptor complexes that undergo endocytosis and subsequent degradation cetuximab, necitumumab, panitumumab, and matuzumab are representative (46) (**Figure 5**). Two phase III studies have evaluated the effect of combining cetuximab along with platinum doublet chemotherapy to treat advanced NSCLC and exhibited a slight improvement in median OS (11.3 months with cetuximab vs. 10.1 months without cetuximab) (4, 48).

Currently, necitumumab is being studied in two phase II clinical studies: INSPIRE on non-squamous NSCLC and SQUIRE on squamous NSCLC to evaluate cisplatin-gemcitabine in combo with necitumumab. From the SQUIRE study, an improved OS was observed. Panitumumab and matuzumab are other mAbs currently in phase II trials (4). Monoclonal antibodies also exert immunologic mechanism inducing ADCC (antibody-dependent cellular cytotoxicity) (49).

Necitumumab is anti-EGFR recombinant mAb and induces fewer allergic reactions due to the absence of murine systems and induction of antibody-dependent cell-mediated cytotoxicity (ADCC) in most cancer cells expressing EGFR (8). Antitumor effect of necitumumab was studied by Topper et al. as monotherapy and in combination therapy, the latter showing synergistic anti-tumor effects (50) and higher toxicity profile than other EGFR-directed monoclonal antibodies.

## Targeted therapy efficacy

Osimertinib is a kinase inhibitor, which falls under this category of targeted therapy as it targets EGFR gene, thereby halting carcinogenesis in NSCLC patients with a mutation in EGFR gene. The side effects of osimertinib include nausea, vomiting, abdominal pain, decreased blood counts, and rarely cardiotoxicity. However, these symptoms disappear upon cessation of drug therapy (51). In the IPASS trial, non-smokers were randomly assigned to receive the EGFR inhibitor gefitinib or carboplatin with paclitaxel (CP) that reflected a better and superior PFS (progression free survival) in the gefitinib as compared to the later (HR 0.74; 95%, CI: 0.65–0.85; P < 0.0001) and ORR (43% vs. 32.2%; P = 0.0001). The OS was observed as median 18.6 and 17.3 (13). The patients with mutated EGFR showed better response from gefitinib with a 51% reduction in progression (HR 0.48; P < 0.0001). The patients who do not carry a mutated EGFR show a better response to chemotherapy (P < 0.0001) (13).

## Approaches of resistance to targeted therapy

**Figure 6:**
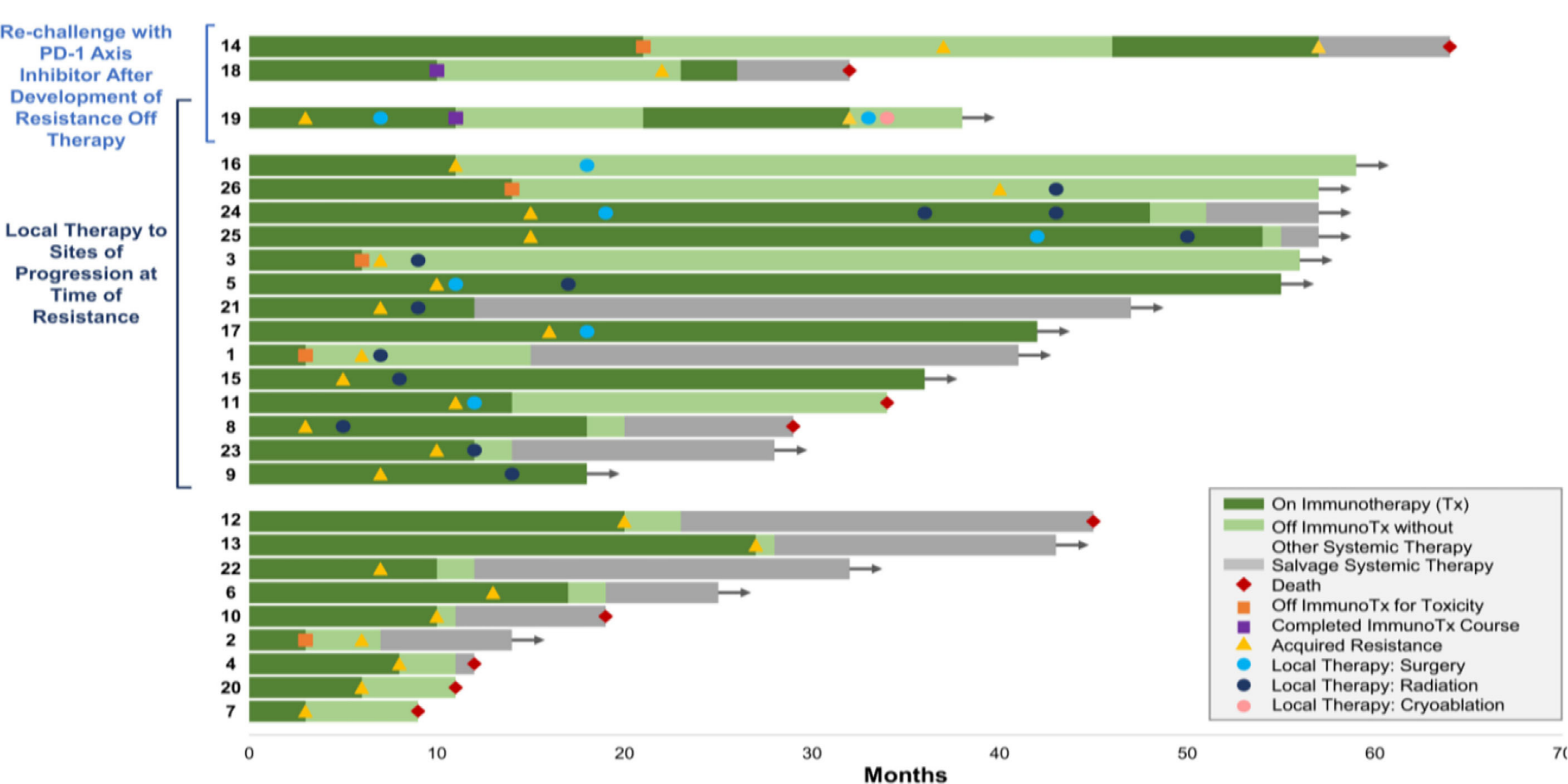
Treatment and resistance (52).

**Figure 7:**
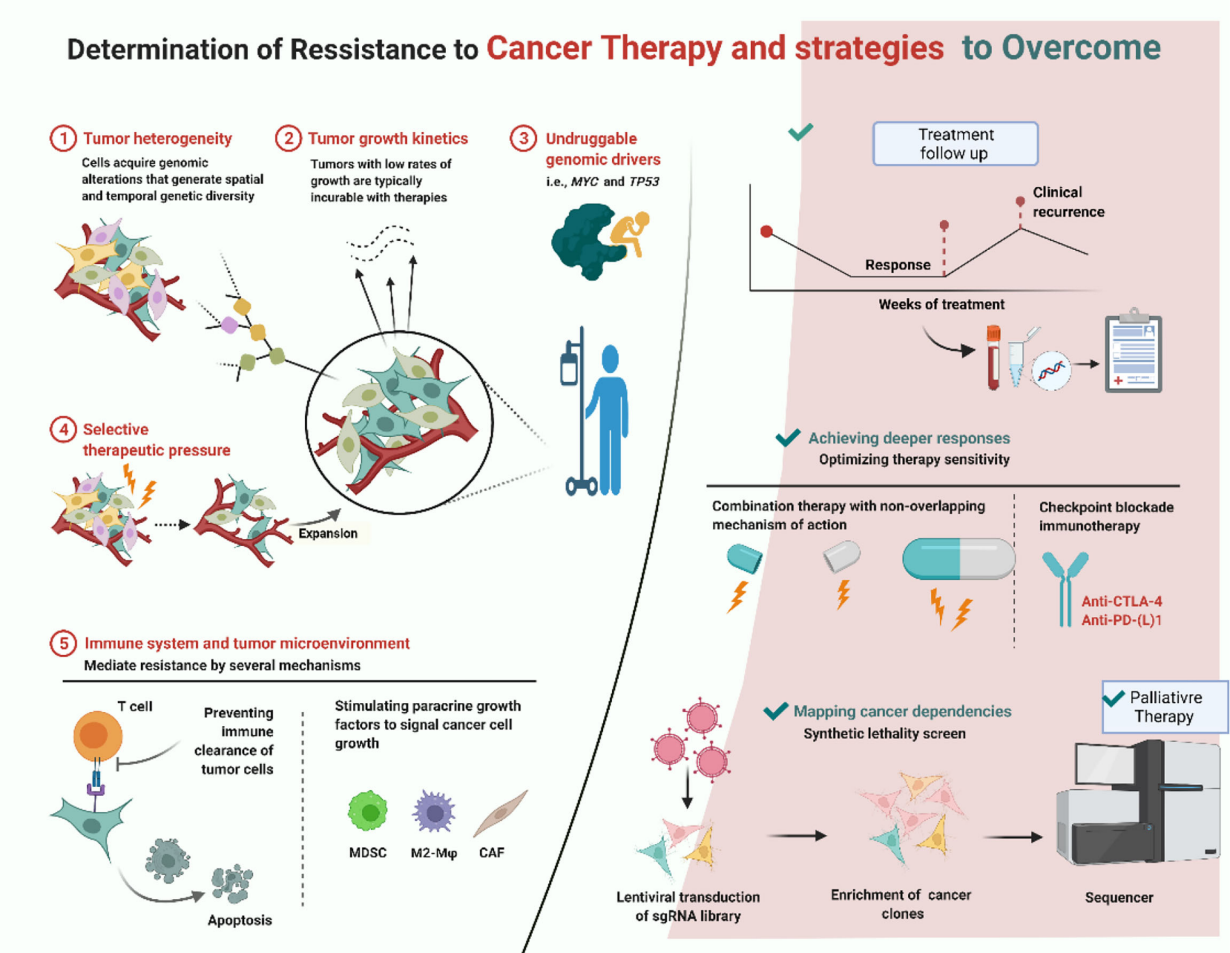
Stages of resistance development and therapeutic strategies.

Despite the fact that EGFR TKIs have dramatically improved treatment approach for EGFR-mutant NSCLC, most responses in many patients do not withhold after 7–12 months. Resistance can develop *de novo* or after body’s exposure to targeted agents and can thrive as resistant clones, both within the same tumor or in different ones in the same patient. Most patients get acquired resistance either by EGFR mutations that follow a primary mutation or *via* activation of EGFR-independent pathways (**Figure 6**). The mechanism of resistance for EGFR activation includes increased EGFR expression and increased subsequent ligand production on malignant cells and, lastly, the presence of mutations of EGFR in malignant/tumor cells. EGFR is a primary therapeutic target and, currently, it is inhibited by TKI and a targeted monoclonal antibody both reversibly and competitively inhibits the ATP for tyrosine kinase domain of EGFR which inhibits all resultant downstream pathways. The EGFR incidence of mutation is high in Asian: up to 50% adenocarcinomas bearing EGFR mutations (**Figure 7**).

Therefore, it is advised to re-biopsy a patient as disease progresses to assess latest tumor biology. In about 50% of resistance cases, basic mechanism is developing a mutation in exon 20 of EGFR which codes for T790M. As a result, methionine replaces threonine and thereby changing kinase domain’s configuration and increasing its affinity for ATP, as compared to wild-type, and decreasing its affinity for first-generation TKIs (4). Another mechanism, which is present in 5%–20% of cases, is based on amplification of MET to overcome EGFR inhibition *via* PI3L-AKT-mTOR signaling. Other resistance mechanisms comprise mutations at PIK2CA, HER2, BRAF, STAT3, AXL kinase, and CRKL amplification. A transformation into small cell lung cancer is also observed in 5% of cases. However, empirical cytotoxic chemotherapy still holds as the treatment of choice because about 30% of resistance has unknown mechanisms (4). The targeted therapy focusing on EGFR tyrosine kinase is used to treat lung cancer (10%–20%) but resistance develops due to mutations (53).

For advanced NSCLC, the NRF2 or NEF2L2 is important in cancer advancement (54), metastasis, and exhibiting resistance to immunotherapy (55). NRF2 is usually exploited by way of most cancer cells in order to lessen oxidative strain and perhaps lead to chemo-resistance. One of the strategies is to target NRF2 and its downstream molecules as interfering with most cancer metabolism, including glutaminolysis and fatty acid synthesis.

Similarly, TP53 tumor suppressors are the most abundant mutations genes and can cause resistance (56). An NRF2 activation is an extraordinary event in EC, related to NFE2L2 or KEAP1 mutations that studies clinical benefits provided by large-scale adoption of molecular profiling in lung cancer.

Screening is essential to routinely assess cancer. The molecular screening, involving the largest sample 17,664 patients within advanced-stage NSCLC patients [71], enabled detection of at least one actionable molecular alteration in almost 50% of analyses and affected treatment plans for 51% of patients. Improving median standard survival was 4–7 months longer without causing genetic mutation.

Among one of the studies in 37 patients with drug resistance in NSCLC is through either EGFRT790M or MET gene amplification. Resistant cancer occasionally reflects gene mutation and amplification through gene of PIK3CA or epithelial cells leading to mesenchymal transition. In the study, 14% of tumors were sensitive to standard treatments since transformation from NSCLC to SCLC. The selective pressure of EGFT inhibitor treatment [imatinib (Gilotrif), dacomitinib (Vizimpro), entrectinib (Rozlytrek), erlotinib (Tarceva), gefitinib (Iressa), and osimertinib (Tagrisso)] led to resistance and genetic mutations (57).

It is important to first identify aberrant pathways. In order to quickly identify significant mutations and resistance mechanisms at tumor tissue and circulating tumor DNA, next-generation sequencing is performed with advanced NSCLC patients. Reducing DNA repair increases the sensitivity of treatment in case of drug resistance to platinum-based chemotherapy and vice versa. Variations at ERCCI and ERCC2 enhance response to platinum chemotherapy but their overexpression will reduce patient survival with gemcitabile-cisplatin treatment.

**Figure 8:**
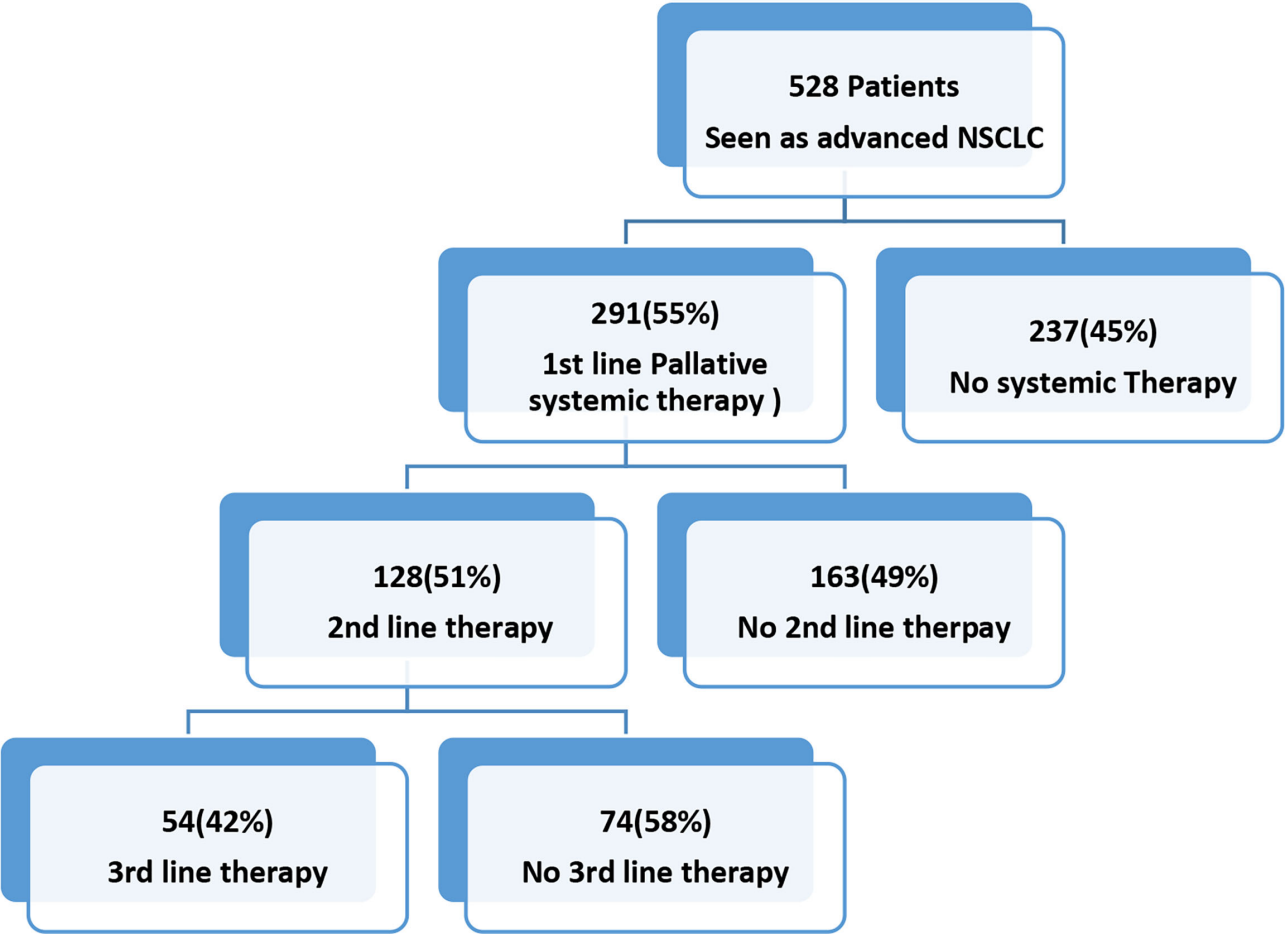
Systemic therapy uses in patients with advanced NSCLC (59, 60).

PD-1 ligand inhibitors develop resistance with approximately 88% recurrence, and 58% of patients were treated with local therapy in contrast to systemic therapy where improved survival rate was observed for 2 years (58). Long-term survival in some patients referred as LTLC may occur due to invasive procedures like lung resection, RT, and differential chemotherapy, leaving patients with high risk of disease reoccurrence and developing comorbidities (1) (**Figure 8**).

## MET inhibitors

The advanced chemotherapy for tumors exhibiting MET exon 14 skipping mutations presents with a good therapeutic outcome. Mechanism involves activation of oncogenic driver MET protein and reducing degradation. In 2020, FDA has approved lapatinib, an inhibitor of MET protein for adult therapy, and is preferred over chemotherapy and immunotherapy. Another inhibitor, tepotinib, is underway for conditional approval after confirmatory trial response (1).

## RT combined with chemoradiation to treat locally advanced NSCLC

The two important aspects to remember when treating locally advanced NSCLC include the effect of local tumor control on the OS in patients at risk for metastatic spread and the toxic effects of radiation on chest hosting extensive tumor growth, and, whether or not, a high-dose RT would improve patient’s survival rate and quality of life (61). An analysis of 11 RTOG trials consisting of 1,356 patients demonstrated a 2-year survival rate of 38% and 5-year survival rate of 15% with chemoradiation (62). The local failure rate (LFR) was reported to be 46% and 52% for 2 and 5 years, respectively.

Chemoradiation consisting platinum-based chemotherapy concurrent with radiation remains the standard protocol for locally advanced-stage NSCLC; however, local tumor control and OS are poor. Socinski et al. reported initial local failure in 46% of patients following neoadjuvant and concurrent chemotherapy (63). Overall, an escalation in radiation dose is believed to improve local control and OS in stage III NSCLC patients according to non-randomized trials and a secondary analysis of RTOG of over 1,300 patients undergoing chemoradiation (64). Genomic profiling

Current advanced treatment of NSCLC is being guided by genomic profiling and genotyping, providing efficient information on fundamental biological and molecular mechanisms, confirming multiplexity of NSCLC. It led to adjustments in the treatment selection, based on pharmacologic and clinical outcome selection of biomarkers based totally on molecular profile (65). The existence of genomic alterations and tumor suppressor genes has arisen as principal precept and pattern can capture complexity regarding tumor development, metastasis, immune microenvironment, and therapeutic susceptibility to TP53 and NFE2L2.

## Advanced strategies

A limited portion of patients with NSCLC responds well to immunotherapy. Biologics therapy also called immunotherapy uses interleukin-2 (IL-2) and is in current medical expertise (66) being the standard care for advanced NSCLC along with monoclonal antibodies addressing the need of treatment by improving response and survival of NSCLS patients (66). Formerly unanticipated long-term responses in advanced stages of NSCLC have been done, with 5- to 12-month OS of 20%–40% in unselected versus patients expressing high PD-L1 levels (67).

The other advanced therapies include the use of checkpoint inhibitors for PD-1 and CTLA-4 pathways. Examples are pembrolizumab, nivolumab, and ipilimumab. Pembrolizumab is advanced first-line treatment for advanced-stage NSCLC and more than 50% of cells express PD-L1 in those cases where driver mutations are non-existent (68). Targeted treatments through tyrosine kinase and ICI are recent advances (69). Treatment with pembrolizumab improved RR (45% vs. 28%), PFS (10.3 vs. 6 months; P <.001; 95% CI, 0.37–0.68; HR, 0.50), and OS (30 vs. 14.2 months), making pembrolizumab as the standard care for these types of patients (19). The targeted therapy with IL-2 is administered orally as well as intravenously.

Some TKI molecules poziotinib, pyrotinib, and mobocertinib have been studied to improve their effect on NSCLC. Furthermore, a currently posted ADVERT HOC, a secondary analysis *(*LUX-Lung 8 trial), has discovered that position of ERBB mutations is among vital biomarkers, in particular HER2 mutations (49).

In a recent study, mutation in TP53 and KRAS resulted in better response to immunotherapy and efficacy in NSCLC and improved PFS as compared to without co-mutations (70). The environmental factors of smoking with BMI and the presence of expression of estrogen receptor in epithelial cells are key regulators for mutation.

## Precision treatments

Another targeted therapy for NSCLC is use of RET inhibitors, i.e., pralsetinib and selpercatinib, gaining recent approval from FDA, for adult treatment instead of immunotherapy and/or chemotherapy. Serious toxicities, 45%–58% for both drugs, include hypertension, high-level aspartate aminotransferase, hyponatremia, and lymphopenia (71). But numerous critical parameters are crucial for consideration, i.e., inadequate response, resistance to pralsetinib and selpercatinib (≈^1^/_3_ of RET-altered cancers), and acquired resistance to RET TKIs *via* secondary on-target and/or driver mutations. Mutation burden of tumor with excessive TMB, with accompanying elevated neoantigen expression, performs a crucial position in antitumor immunity. The following are illustration development and control of obtained resistance to programmed loss of life with axis inhibitor therapy., 7

## Improvement in survival rate

Twenty-six percent of all patients with NSCLC live ≥ 5 years after diagnosis (72). The annual survival rate of NSCLC has been improved from 2.4% to 5% overall, while simultaneous incidence has been reported to decrease (2.2%–2.3%). The comparisons were made for two-drug and three-drug regimens for chemotherapy and the latter proved significant benefit in progression-free survival. The improvement in trends from 1.8% to 4.4% in women and 3.1% (2009–2013) to 5.5% (2014–2018) in men has been reported and was distinct in women and all races and ethnic groups (73). Visual decline in lung cancer mortality doubled (from 3.1% from 2009 to 2013 to 5.5% from 2014 to 2018) in men and (1.8%–4.4%) in women with 2.4%–5% overall decline. This trend coincides with steady declines in occurrence (2.2%–2.3%) but rapid gain in survival in NSCLC.

The relative survival rate in NSCLC increased from 34% to 42% from 2009 to 2016 with an estimated 6% for each stage of lung cancer attributed to targeted therapy, while, at the same time, survival of SCLC remained 14%–15%. Therefore, there is a decrease in overall mortality to 3.1%. The studies revealed an improved disease prognosis with a patient exhibiting BRAF V600 E mutations and an improved OS rate (3 years). This contrasted with patients without RAF V600 mutations (24%) (73).

For instance, 2-year NSCLC relative survival rate increased from 34% to 42% (2009 and 2010–2015 and 2016, respectively), including absolute increase of 5%–6% at every stage with only 14%–15% survival for small cell lung cancer patients. Improved treatment showed excellent response against lung cancer and provided record decline in overall cancer mortality (74).

BRAF mutation in NSCLC exhibits less therapeutic improvement but appears to be responsive to immunotherapy due to aggressive clinical features of three distinct functional classes. The evidence did not exist for combination therapy explaining the use of BRAF or MEK inhibitors against non-V600E BRFA mutant NSCLC (73).

The sotorasib is among the targeted therapeutic agents approved by US FDA for NSCLC of local and advanced metastatic lung cancer with KRAS mutations (75). The drugs exhibit extensive adverse drug reactions. In a recent study, 88% improved response rate in NSCLC was observed with sotorasib with PFS of 6.3 months and less than 5% adverse effects each in LFT abnormalities, diarrhea, anemia, hepatitis, and hyponatremia (76). Another related molecule is adagrasib having a 45% response rate. The resistant refractory to other standard therapy is treated preferably by trastuzumab-based regimen, e.g., trastuzumab-druxtecan, showing 55% response rate and PFS of 8.2 months with 17.8 months of OS (77).

Any significant association between HER2 mutations and HER2 amplification could not be found. Initial clinical studies exhibited no results for targeted therapy in HER2-amplified NSCLC. Interim analysis of DESTINY-Lung-01 study demonstrated 24.5% response rate using various genotypes such as P13K and CTNNB1, and also tumor suppressors STK11, KEAP1, and NFE2L2. Alterations in genes do not lead to sensitivity to the targeted therapy. The STK11 alterations demonstrate relative resistance to immunotherapy and KEAP1 mutations increase resistance to radiotherapy (78). Siglec-15 antibody is an immunoglobulin-like protein in lots of human cancers that works as critical immune suppressor and is, at the same time, unique to PD-L1 (79).

## Palliative chemotherapy and outcomes

Palliative chemotherapy is directed to enhance the quality of life and survival; however, some patients still remain untreated (59). Studies suggest not a great fee of development in survival through the use of aggregate therapies. The palliative care of NSCLS is focused on provisions of suitable treatments and symptomatic treatment of pain, dyspnea, nausea/vomiting, and fatigue. The chemotherapeutic drugs lead to pulmonary toxicity and require management in palliative care (59). Still, a fragment of patients of advanced NSCLC gets hold of any form of systemic treatment.

Palliative treatment options for endobronchial tumors include chemotherapy, radiotherapy, endobronchial laser resection, or stent placement. As a rule, cough improves if directed therapy reduces impact of cancer. However, symptom improvement with endobronchial brachytherapy, radiotherapy, or palliative chemotherapy can take multiple weeks. Mild cough options include patient counseling, use of linctus such as honey, cough suppression techniques, and/or breathing exercises. If they are useless, patient is then prescribed peripherally performing antitussive (e.g., benzonatate). If symptoms do not improve with a peripherally acting antitussive, a centrally acting antitussive is indicated, as for patients with a more severe cough.

The nebulization with lidocaine and bupivacaine is also used in serious cases for specialized palliative care clinics. There has been limited efficacy of dextromethorphan as a cough suppressant in cancer patients. Opioids are first-line treatment for palliative care patients with severe cough with intrathoracic cancer. Addiction is a rare concern. The opioids used are morphine, codeine, and dihydrocodeine. Higher efficacies were observed at high doses. The patients already receiving opioids are being prescribed with 25%–50% higher dose than the current dose to alleviate symptoms. Morphine is the preferential treatment of choice (80). Due to development of P450 cytochrome enzyme, the Asian population are at greater risk of developing codeine adverse effects.

The evidence from trial indicates that opioids reduce cough severity and frequency to improve quality of life. The monitoring is done for sedation which declines after 1–3 days. Other side effects are peripheral edema, weakness, nystagmus, nausea, somnolence, tremor, and emotional lability. Gabapentin has been used to relieve cough refractory to gastroesophageal reflux with dose of 300 mg/day to reduce occurrence of sedation and dizziness. Adjunctive therapies in palliative care include expectorants for thickening of sputum. Examples include guaifenesin and nebulized nasal saline and acetylcysteine as mucolytic.

Bronchospasm symptoms are treated with ipratropium bromide and inhaled ibuterol. Pharmacologic therapies for excess secretions include anticholinergics and most used are intravenous preparations of glycopyrrolate. Glycopyrrolate is also given subcutaneously and sublingually to reduce excess secretions.

Hemoptysis is frequently observed in patients with lung cancer due to elevated bronchial secretions. Approximately 20% NSCLC patient exhibit hemopytysis at any stage in life of cancer patients exhibit hemoptysis at any stage in life. Palliative treatments of hemoptysis include management of bleeding: use darker shades of accessories (such as towel, dressings, sheets, blankets, and absorptive dressings), avoid using white cups at bedside and red-streaked white tissues and environmental management. The management of life-threatening hemoptysis is adjusting position of patients to prevent non-bleeding lung from spillage of blood, which can cause blockage of gases with clots or filling alveoli with blood. Supportive care with blood and platelet transfusions is administered for reversal of anticoagulation and administration of procoagulant.

Therapeutic bronchoscopy performed by balloon tamponade and infusion of adrenaline is successfully used. Oral and nebulized antifibrinolytics are used. Nebulized vasopressin and tranexamic acid have been reported with response rates of 60%–100%. Nebulized tranexamic acid has been helpful in case reports. If the area of bleeding is directly visualized, bronchoscopy techniques (laser coagulation/electrocautery) may be used with response rates of 60%–100%. Numerous phase III clinical studies proved that palliative strategy for advanced NSCLC may improve outcomes. This includes extended survival and enhanced life quality with lung cancer prevalence of 11.4% (81).

## Complications

Complications of treatments are enhanced with aged and medically ill patients. The aged patients are the majority among NSCLC. Furthermore, malnutrition and depression have been reported and associated to increased mortality, indeed in aged patients, with progression-free tumors with less adherence to treatment and poor lungs performance (82). Definitive radiotherapy is advised as a suitable choice of treatment for aged patients (≥75 years) with inoperable or unresectable NSCLC. The consecutive chemoradiotherapy or radiotherapy alone is applicable for senior patients. Still, increased toxicity is a consideration. Retreatment after initial response led complications of colitis (17%), rash (16%), pneumonitis (19%), and liver enzyme abnormalities (10%). Retreated patients had resolution of irAEs or improvement to approximately grade 1 in comparison to those with discontinued treatment (97 vs. 76, P = 0.01). Overall, among the 48 patients, exhibited PFS and OS improved with retreatment. The retreatment with ICI led to grade 3 or grade 4 toxicity (83). Relapse after definitive remedy may pose predominant patterns of failure, making an argument for chemotherapy either sequentially or concurrently (84).

## Local management of metastasis

Advancements in OS with multimodality regimens, i.e., chemotherapy with surgery and/or radiation, have shown decreased prevalence in preventing brain metastases of advanced NSCLC. Locally advanced NSCLC poses greater threat in development of brain metastases. It may identify a definite group of patients, benefiting from aggressive management strategies, to address this issue after completion of local therapy (85). Avoidance of EGFR impediments for patients with EGFR wild type/mutated NSCLC is normally characterized by “uninflamed” tumor microenvironment, weak immunogenicity, and immunological tolerance (86).

## Conclusion

In this review, we have discussed the latest staging and treatment strategies of advanced-stage NSCLC. The NSCLC treatment has gained great concern in modern research due to multiple problems faced during the treatment including diagnosis of stage and resistance to conventional therapy. Different therapies have been utilized to effectively treat NSCLC like platinum-based chemotherapy, chemo-immunotherapy, and, most importantly, the targeted therapy. NSCLC if diagnosed and treated at early stages can be treated effectively; materials were discussed in our review. Furthermore, due to unique effects of chemo-immunotherapy and targeted therapy, the occurrence of disease has been improved in many studies. Moreover, NSCLC treatment strategies need to be further investigated to establish safe and effective treatment options without resistance being caused.

## Author contributions

RF, AZ, YY, and MR contributed to the conceptualization of the study. The design by RF, AZ and MR. Data collection by TH, KS analysed and interpreted the data. RF and AZ wrote the first draft of the manuscript. YD and MR revised the article for important intellectual content. All authors read and approved the final manuscript.

## Conflict of interest

The authors declare that the research was conducted in the absence of any commercial or financial relationships that could be construed as a potential conflict of interest.

## Publisher’s note

All claims expressed in this article are solely those of the authors and do not necessarily represent those of their affiliated organizations, or those of the publisher, the editors and the reviewers. Any product that may be evaluated in this article, or claim that may be made by its manufacturer, is not guaranteed or endorsed by the publisher.
